# Botanical Interventions to Improve Glucose Control and Options for Diabetes Therapy

**DOI:** 10.1007/s42399-021-01034-8

**Published:** 2021-08-15

**Authors:** Peter Smoak, Susan J. Burke, J. Jason Collier

**Affiliations:** 1 Laboratory of Islet Biology and Inflammation, Pennington Biomedical Research Center, Louisiana State University System, 6400 Perkins Road, Baton Rouge, LA 70808, USA; 2 Immunogenetics Laboratory, Pennington Biomedical Research Center, Louisiana State University System, 6400 Perkins Road, LA 70808 Baton Rouge, USA

**Keywords:** Botanical, Diabetes, Islet, Obesity, Inflammation

## Abstract

Diabetes mellitus is a major public health problem worldwide. This endocrine disease is clustered into distinct subtypes based on the route of development, with the most common forms associated with either autoimmunity (T1DM) or obesity (T2DM). A shared hallmark of both major forms of diabetes is a reduction in function (insulin secretion) or mass (cell number) of the pancreatic islet beta-cell. Diminutions in both mass and function are often present. A wide assortment of plants have been used historically to reduce the pathological features associated with diabetes. In this review, we provide an organized viewpoint focused around the phytochemicals and herbal extracts investigated using various preclinical and clinical study designs. In some cases, crude extracts were examined directly, and in others, purified compounds were explored for their possible therapeutic efficacy. A subset of these studies compared the botanical product with standard of care prescribed drugs. Finally, we note that botanical formulations are likely suspects for future drug discovery and refinement into class(es) of compounds that have either direct or adjuvant therapeutic benefit.

## Introduction

Diabetes mellitus describes a cluster of endocrine diseases that are sub-classified based on the etiology of development. Type 1 diabetes mellitus (T1DM) is proposed to occur via autoimmune mechanisms targeting the pancreatic islet beta-cell, leading to reduced circulating insulin [1]. Type 2 diabetes mellitus (T2DM) is most commonly associated with obesity as a major risk factor, which leads first to insulin resistance and then to eventual beta-cell failure [2]. Thus, both T1DM and T2DM have in common a reduction in the mass and/or function of the pancreatic islet β-cells [3, 4]. Collectively, all forms of diabetes mellitus create numerous clinical problems, including diseases of the eyes, kidneys, and cardiovascular system [5]. As of 2019, it is reported that 8.8% of the global population (representing greater than 460 million people) have diabetes; this unfortunate reality places an enormous burden on the healthcare systems of the individual countries [6]. In addition, and perhaps not surprisingly, over the past decade, diabetes has consistently been one of the top 10 leading causes of death worldwide.

T1DM accounts for 5–10% of all diabetes mellitus cases and has historically been thought to affect children more often than adults [7]. However, more recent evidence shows that T1DM can also affect adults over 18 at almost equal incidence to children [8]. T2DM is the most common form of diabetes. This form of the disease is closely linked to overweight and obesity, chronic inflammation, insulin resistance, and ultimately islet β-cell de-differentiation, death, and/or dysfunction [9–11]. In healthy individuals, islet β-cells in the pancreas produce and secrete insulin, a hormone responsible for the metabolism and partitioning of macronutrients [12]. However, when insulin production and secretion are insufficient, alterations in metabolism occur and accumulation of glucose in the blood signals the clinical diagnosis of diabetes [13–15]. In addition to elevations in blood glucose, T2DM has been linked with alterations in blood lipid concentrations, oxidative stress in various tissues, and changes in endoplasmic reticulum function [16, 17]. Despite the cause or type of diabetes, it is evident that maintaining islet β-cell function, enhancing β-cell replication capability, and protecting against losses in total β-cell mass are important factors for the control and prevention of these endocrine diseases [3, 18–20].

The search for improved diabetes treatments is critically important due to the global increase in incidence and prevalence of the major forms of diabetes. One such strategy being actively considered is the use of plant-based derivatives (often a mixture of complex molecules) to improve blood glucose control. Along these lines, botanical products have historically been used to treat a plethora of human diseases and chronic ailments, including diabetes. Some of the documented treatments, which were initially without mechanistic targets, eventually led to the development of commonly used anti-diabetic drugs. For example, metformin, one of the most widely used insulin-sensitizing pharmaceuticals on the market, arose from studies on the plant *Galega officinalis*, also known as French lilac. The active biguanide compound was later isolated and synthesized into dimethylbiguanide, which is now sold as metformin [21]. In addition, combinations of herbal extracts contained within a single formulation are also being investigated in the hopes that individual botanicals will synergize to provide greater efficacy compared with a single extract [22].

Many additional botanicals are being tested in the hope that they too will yield promise as plausible and viable options to treat symptoms or reduce the deleterious consequences of diet, genetics, or environmental factors that contribute to the progression to diabetes. It is conceivable that many of these natural bioactive compounds will be modified using medicinal chemistry approaches to enhance their potential as novel pharmaceuticals. As many as 50% of currently used drugs are natural bioactive compounds or derivatives of such compounds [23]. In this review, we will focus on studies of botanical compounds that have reported beneficial properties relevant to pancreatic islet β-cells in the context of either obesity, T1DM, or T2DM.

## Methods

A literature search was performed using PUBMED and Google Scholar as the primary search engines for the following keywords: anti-diabetic, β-cell function, β-cell proliferation, β-cell inflammation, and pancreatic inflammation with no year restriction. From the results of this database query, articles retrieved from English language journals were screened for reported use of botanicals. Studies that also described the isolation of the active components identified within the experimental substances were included. We focused particular attention on the specific impact of such botanical-based approaches to impact pancreatic β-cells either in vitro or in vivo. Collectively, this literature search formed the basis of this integrative narrative review.

## Results

Although numerous studies have explored the effects of natural bioactive compounds for their anti-diabetic properties, a frequently encountered limitation is the study of the broad anti-diabetic effects of a plant extract without isolating the key active compounds. In many cases, the active component(s) are not known. In this review, we focus our attention on a variety of botanicals that report a beneficial impact on pancreatic β-cells. The parameters addressed herein include insulin secretion, markers of β-cell death, inflammation and related signaling pathways, and changes to β-cell replication or other measures that would lead to enhanced cell numbers.

## Berberine

Berberine is an isoquinoline alkaloid isolated from roots, rhizomes, stems, and bark in several plants including *Berberis*, Amur cork trees, and California poppy (Table 1). The use of plants containing berberine for treatment purposes dates back to as early as 650 BC, where it was used for the treatment of inflammation, infectious disease, wounds, and many other ailments [24]. In more recent times, berberine has been shown to have protective effects against a number of diseases including liver injury, cancer, cardiovascular disease, and neurological disorders, due to anti-oxidant, anti-proliferation, and anti-ischemic properties [25, 26].

Using rodent models of obesity and T2DM, treatment with berberine has been shown to improve fasting blood glucose (FBG), glucose tolerance, and fasting insulin levels [27–29]. Additionally, berberine has been reported to protect against streptozotocin (STZ)-induced β-cell death [30, 31]. Furthermore, in patients with T2DM, long-term treatment with berberine proved to have similar efficacy as the commonly prescribed anti-hyperglycemic agent metformin [32].

### In Vivo Studies

Administration of berberine (5 mg/kg/day) via intraperitoneal (i.p.) injection to 12-week-old *db/db* mice, a model of human T2DM [33], resulted in reduced FBG and improved glucose tolerance. This was accompanied by a 13% weight loss when compared to *db/db* mice administered a vehicle control, which may have contributed to the improved glucose control. Importantly, berberine treatment did not alter food intake, yet weight loss occurred via a reduction in epididymal fat mass relative to the vehicle control group [27].

In Wistar rats fed a 60% high-fat diet, berberine supplementation (380 mg/kg/day, oral gavage for 2 weeks) improved glucose tolerance, decreased insulin resistance, and reduced plasma triglyceride (TG) concentrations in the high-fat diet (HFD) group compared to the vehicle-treated rats [27]. Whether the improvement in glucose tolerance was directly induced by berberine or a result of weight loss was not addressed. Similar improvements in glucose tolerance, insulin sensitivity, and plasma triglycerides were seen in high-fat-fed Sprague Dawley (SD) rats administered berberine (150 mg/kg/day, oral gavage) for 6 weeks [28].

In Wistar rats fed a 60% high-fat diet for 6 months and then given berberine supplementation (125 mg/kg/day, oral gavage twice daily for 5 weeks), a clear decrease in both fasting and post-prandial blood glucose levels was seen during an ipGTT compared to high-fat-fed control animals. Fasting insulin levels were also reduced in the berberine-treated rats [29].

A dose-dependent improvement in glucose tolerance was observed following a 4-week treatment with two doses of berberine [187.5 and 526.5 mg/kg/day, intragastrically (i.g.)] in Wistar rats given a single i.p. injection of STZ (30 mg/kg)] [30]. Fasting insulin levels in the rats given the highest dose of berberine were significantly different from control animals. Furthermore, FBG levels and plasma TG were significantly reduced compared to STZ-injected animals without berberine supplementation. In this study, the highest dose of berberine was shown to be as efficacious as metformin in lowering glucose and insulin levels compared to STZ-treated animals without intervention [30].

In rats administered a single 35 mg/kg injection of STZ in combination with a high-fat/high-sugar diet, 16 weeks of berberine treatment (150 or 300 mg/kg/day, via gavage) decreased serum insulin levels and improved insulin sensitivity relative to rats without a drug regimen. A similar outcome was seen in animals treated with rosiglitazone (PPARγ agonist), but not fenofibrate (PPARα agonist). Furthermore, both berberine and rosiglitazone, but not fenofibrate, protected against loss of insulin-positive cell mass in STZ-injected diabetic rats. Berberine also protected against STZ-dependent β-cell damage, improved pancreatic superoxide dismutase (SOD) abundance, and reduced pancreatic malonaldehyde to similar values as the non-diabetic group [31]. Increased survival and function are also thought to be a result of the improved anti-oxidant defense. Berberine increases anti-oxidant enzymes like SOD, helping to explain the reduction in oxidative stress, resulting in improved β-cell integrity and decreased loss of function [31].

Berberine was also shown to be effective in human studies. In a randomized controlled trial, male and female patients with T2DM treated with berberine (1500 mg/day, oral tablet) for 3 months showed similar improvements as metformin on lowering FBG, post-prandial blood glucose (PBG) concentrations, and HbA1c. As part of a combination therapy with metformin, improvements were seen in fasting and post-prandial insulin values. In addition, there was an improvement in HOMA-IR as early as 5 weeks in berberine- and metformin-treated patients. A limitation of this study is that HOMA-IR was only measured as a combination therapy with metformin and not when using either drug as a monotherapy [32].

### In Vitro Studies

Despite the benefits observed in vivo, the effects of berberine in vitro are conflicting, specifically with insulin secretion. The effects of berberine on insulin secretion seem to be dose-dependent; however, whether it increases or decreases insulin secretion is not clear based on existing studies.

In hamster HIT-T15 cells, overnight treatment with berberine (10 μM) in the presence of increasing concentrations of glucose potentiated insulin secretion. In addition, berberine (1 and 10 μM) potentiated glucose-stimulated insulin secretion in a dose-dependent manner. Interestingly, 100 μM berberine was shown to be toxic to HIT-T15 cells [30].

However, the insulinotropic effects of acute treatment of insulinoma cells with high berberine concentrations are uncertain. Two studies using similar cell lines and methods produced contradictory results. Acute treatment of mouse MIN6 cells with berberine (1–50 μM for 1 h) reduced insulin secretion in a dose-dependent manner. Treatment of MIN6 cells with berberine (2.5 μM for 1 h) also reduced palmitate-dependent potentiation of glucose-stimulated insulin secretion [28]. Conversely, in a separate study, MIN6 cells acutely treated with berberine (50 μM for 30 min) increased insulin secretion in a dose-dependent manner [34]. Perhaps limiting the time of cell exposure to high doses of berberine may be important in eliciting the insulinotropic effects. Nevertheless, more evidence is needed to fully elucidate the effects of berberine in vitro.

Mechanistically, activation of AMPK is thought to be at least partially responsible for the increased insulin secretion in response to berberine seen in some studies. Berberine promotes phosphorylation of AMPK in 3T3-L1 adipocytes [27, 29] and primary mouse adipocytes [27]. In L6 myotubes, berberine promotes phosphorylation of AMPK (Thr^172^) [27, 29] and translocation of GLUT4 to the plasma membrane [27]. In primary human and rodent islets, pharmacological activation of AMPK under glucose stimulatory conditions has been shown to potentiate insulin secretion due to an increase in intracellular Ca^2+^ (_i_Ca^2+^) concentrations [35]. Despite the clear effects of berberine on activation of AMPK in adipocytes and myotubes, the direct ability of berberine to activate AMPK in either primary islets or β-cell lines has not been tested.

## Capsaicin

Capsaicin is a major active component of chili peppers and is the predominant capsaicinoid found in any fruit belonging to the *Capsicum* genus [36, 37] (Table 1). Capsaicin was first extracted in 1816 and called capsicin [38]; however, it was not until 1876 when capsaicin was first truly isolated [39, 40]. Capsaicin has been reported to have beneficial roles in treating obesity, cardiovascular, gastrointestinal conditions, various cancers, neurogenic, and dermatologic conditions [41]. Administration of capsaicin has also been shown to have anti-hyperglycemic effects in a number of rodent models of obesity and T2DM [42–45]. In certain studies, this observation correlated with an improvement in glucose tolerance and lowering of HBA1c levels, with or without alterations in plasma insulin levels. Anti-diabetic effects were also reported in rodent models of T1DM, including NOD mice, as well as models of acute inflammation: STZ-injected animals, and a partial pancreatectomy study. Further, capsaicin was proven to be efficacious in pregnant women with gestational diabetes mellitus (GDM).

### In Vivo Studies

Supplementation of capsaicin (0.015% of diet) to high-fat diet fed C57BL/6 mice for the last 10 weeks of a 20-week dietary protocol resulted in a reduction in body weight without decreased food intake. This decrease in body weight correlated with a reduction in fasting plasma concentrations of glucose and insulin, improved glucose tolerance, and reduced TG concentrations [42]. Thus, it is possible that capsaicin increases energy expenditure, which has been documented in other studies [46].

KKAy mice, a genetic model of obesity, fed a high-fat diet for 5 weeks were supplemented with 0.015% capsaicin in their diet for the final 2 weeks of the study. Fasting plasma glucose, insulin, and TGs were all decreased in the dietary capsaicin group compared to the control mice; interestingly, these changes occurred without any alteration in body weight [43]. Dietary supplementation of capsaicin (0.0042%) to female KKAy mice for 30 days also significantly lowered glucose levels as early as 19 days into the study, with no observable changes in plasma insulin levels [44].

Zucker diabetic rats, a model of obesity, insulin resistance, and T2DM, were injected with three ascending doses [20, 30, and 50 mg/kg, subcutaneous (SQ)] over 3 days and subsequently monitored for 60 days post-treatment. Despite a larger weight gain in the capsaicin-treated group over 60 days compared to vehicle-treated Zucker diabetic fatty (ZDF) rats, fasting plasma glucose levels remained in the physiological range over the 60-day monitoring period, and HbA1c levels were significantly lower in capsaicin-treated rats at the end of the study. Capsaicin-treated ZDF rats demonstrated improved glucose tolerance without any alteration in plasma insulin compared to vehicle control rats. Furthermore, glucose-stimulated insulin secretion was restored in islets from capsaicin-treated animals compared to a completely blunted response to glucose in ZDF vehicle controls [45]. This phenotype was accompanied by a total loss of TRPV1 expression in islet-innervating fibers of the pancreas [45]. Following initial stimulation, prolonged capsaicin stimulation is known to promote desensitization and deterioration of neuronal fibers [47, 48].

In STZ-exposed SD rats (single STZ injection 60 mg/kg), 28 days of capsaicin treatment (6 mg/kg/day, oral gavage) significantly improved FBG, glucose tolerance, serum insulin, and glycosylated serum protein (GSP). Capsaicin also increased pancreatic mRNA and protein abundance of TRPV1, pancreatic duodenal homeobox 1 (Pdx1), insulin receptor substrate (IRS)1, IRS2, and glucose transporter 2 (GLUT2) [49]. In female NOD mice, a mouse model of T1DM, 20 weeks of capsaicin treatment (50 mg/kg/day, i.p. injection) significantly reduced insulitis compared to vehicle control mice, corresponding with a delay in diabetes development in capsaicin-treated mice [50].

In a 90% pancreatectomy model of diabetes, SD rats fed an HFD (40% fat) supplemented with capsaicin (0.025% of diet) for 8 weeks displayed an increase in fasting insulin levels, as well as a reduction in fasted glucose concentration, compared to pancreatectomized animals receiving HFD without supplementation. Capsaicin-supplemented diabetic rats displayed an improvement in glucose tolerance, increased insulin secretion during a hyperglycemic clamp, and enhanced β-cell mass relative to non-supplemented HFD diabetic animals [51]. In this model, capsaicin-treated islets had an elevated abundance of IRS2 and Pdx1, a β-cell-enriched transcription factor responsible for maintenance of the adult β-cell phenotype. BrdU^+^ cells were also increased in the islet; therefore, increased β-cell proliferation, along with a reduction in markers of apoptosis, would be predicted to promote increased β-cell mass [51].

Capsaicin also appears to have a beneficial effect in humans. In healthy, non-diabetic males and females, a single dose of capsicum (5 g, oral capsule) containing 26.6 mg of capsaicin significantly lowered plasma glucose levels during an oral glucose tolerance test (OGTT) with a concomitant increase in plasma insulin, when compared to the placebo group [52]. Five milligrams of capsaicin per day over 4 weeks in women with GDM improved post-prandial, but not fasting glucose, and insulin levels [53].

### In Vitro Studies

Currently, the literature regarding the mechanisms of action of capsaicin is conflicting. Capsaicin can directly promote insulin secretion from rat RINm5F cells, although toxicity was observed at the highest dose tested [54]. Furthermore, capsaicin was shown to promote an increase in the intracellular calcium concentration in rat INS-1E cells; however, this response was not recapitulated in Wistar rat primary β-cells or human β-cells [55]. It has been proposed that capsaicin mediates its effects through the transient receptor potential vanilloid type 1 (TRPV1) ion channel [56], although capsaicin is not selective for TRPV1 [57].

TRPV1 mRNA expression was observed in rat INS-1, RINm5F cells [54], and INS-1E cells [58], and immunostaining displayed TRPV1 expression in INS-1E cells [55], rat islet endocrine cells [54], but not in β-cells from ZDF rats [45], NOD mice [50], or from isolated human islets or insulinomas [55]. Although capsaicin can increase mRNA levels and protein abundance of the TRPV1 in pancreatic tissue of STZ-injected rats, a model of insulin insufficiency [49], treatment of INS-1E cells with the TRPV1 antagonist, capsazepine, did not alter glucose-stimulated insulin secretion in INS-1E cells [58]. Given the variability in detecting TRPV1 across different model systems, it is unclear at present whether the effects of capsaicin are mediated directly through TRVP1 in the β-cell. However, it appears that the contribution of TRPV1 in modulating insulin secretion in a capsaicin-dependent manner cannot be completely excluded and may be more complex than previously suggested. Instead, it is possible that pancreatic islets do not express TRPV1, but rather are innervated by TRPV1-expressing sensory nerve fibers, which may play a role in the progression of islet inflammation and autoimmune-mediated diabetes [50] and T2DM [57].

## Cinnamaldehyde

Cinnamaldehyde is an aldehyde derived from cinnamon (Table 1). This compound, which was first discovered in 1834, constitutes about 90% of the oil found in cinnamon bark and is responsible for the flavor and odor associated with the spice [59, 60]. Cinnamaldehyde has been reported to have anti-oxidant, anti-inflammatory, anti-cancer, anti-diabetic, and wound healing properties [61].

A number of studies demonstrate the protective effects of cinnamaldehyde against STZ-induced β-cell injury [62–66]. Whereas acute treatment with cinnamaldehyde was shown to lower fasting blood glucose levels, chronic treatment showed a significant improvement in glucose tolerance and restoration of plasma insulin to non-diabetic levels. Moreover, cinnamaldehyde has been demonstrated to mediate anti-inflammatory effects directly on the β-cell [67].

### In Vivo Studies

In Wistar rats exposed to STZ (single injection, 60 mg/kg), treatment with cinnamaldehyde (20 mg/kg/day, oral gavage) for 45 days markedly reduced plasma glucose, increased plasma insulin to near normal concentrations, and reduced TG concentrations [62]. Similarly, oral administration of cinnamaldehyde for 28 days in diabetic Wistar rats (single injection STZ, 50 mg/kg) improved glucose tolerance, increased plasma insulin, decreased FBG, reduced HbA1c, improved malondialdehyde (MDA) concentrations to near normal, and improved glutathione (GSH) concentrations [63]. In the same study, acute (4 h) treatment with cinnamaldehyde (20 mg/kg, single oral gavage) reduced FBG similar to the effect of glibenclamide, a commonly used anti-diabetic medication [63]. Also in Wistar rats exposed to STZ (single injection 60 mg/kg), treatment with cinnamaldehyde (5, 10, 20 mg/kg/day, oral gavage) for 45 days improved plasma glucose in a dose-dependent manner. Moreover, the highest concentration of cinnamaldehyde (20 mg/kg) protected against the STZ-dependent decrease in plasma insulin levels and, similar to glibenclamide, improved pancreatic concentrations of SOD, GSH, glutathione peroxidase (GPx), and catalase (CAT) [64].

In Wistar rats exposed to STZ (single injection, 50 mg/kg), cinnamaldehyde ingestion (20 mg/kg/day, oral gavage) for 60 days was as effective as glibenclamide in reducing FBG and HbA1c compared to untreated diabetic control animals. Both cinnamaldehyde- and glibenclamide-treated rats showed a restoration of serum insulin to a non-diabetic concentration [65]. Cinnamaldehyde was shown to mediate its effects directly on the β-cell as acute treatment (2 mg/mL for 2 h) promoted a greater than two-fold increase in insulin release compared to islets of STZ-exposed rats. A similar effect was seen with glibenclamide treatment [65].

Cinnamaldehyde supplementation (10 mg/kg/day, oral gavage) for 30 days in HFD (20% sucrose and 12.5% fat combined with 67% normal chow) combined with STZ exposure (single injection, 35 mg/kg) in Wistar rats partially restored glucose tolerance to the level of non-diabetic animals and improved insulin sensitivity. Furthermore, supplementation with cinnamaldehyde improved fasting glucose and restored fasting serum insulin levels to that of the control group. Calculations of HOMA-IR and HOMA-β values showed no significant differences when compared with the non-diabetic control group [66]. Protective effects of cinnamaldehyde may be at least in part due to the restoration of insulin signaling (increased expression of IRS1/PI3K/AKT2) in pancreatic tissue of diabetic rats, decreased levels of advanced glycation end-products (AGEs) in serum of diabetic animals, and normalization of serum lipid profiles, in the presence of cinnamaldehyde [66].

### In Vitro Studies

In rat RINm5F cells, cinnamaldehyde pretreatment (5 and 10 μM for 3 h) inhibited a number of inflammatory processes induced by STZ. First, cinnamaldehyde attenuated the STZ-induced phosphorylation of IκB and blocked translocation of NF-κB into the nucleus. Second, cinnamaldehyde inhibited STZ-induced phosphorylation of extracellular signal-regulated kinase (ERK), c-Jun NH2-terminal kinase (JNK), and p38 mitogen-activated protein kinase (MAPK). Consequently, cinnamaldehyde ameliorated STZ-induced NF-κB activity resulting in decreased nitric oxide (NO) production, iNOS mRNA and protein expression, COX2 mRNA and protein expression, and prostaglandin E_2_ (PGE_2_) production [67]. Reduction in inflammation in the presence of cinnamaldehyde correlated with an increase in β-cell survival [67].

Cinnamaldehyde can activate the transient receptor potential ankyrin 1 (TRPA1) [68], which has been shown to be expressed in isolated islets from Sprague Dawley rats and RINm5F insulinoma cells [69]. Activation of this receptor can promote insulin release through increased intracellular Ca^2+^ influx [69].

## Conophylline

Conophylline can be isolated from the leaves of *Tabernaemontana divaricate* using an ethanol extraction procedure [70] (Table 1). Conophylline has been reported to improve non-alcoholic steatohepatitis, cellular neurodegenerative disease, and islet fibrosis [71]. Its biological properties include promoting differentiation of pancreatic β-cells in culture and increasing β-cell mass in vivo following ablation by STZ [72, 73]. Conophylline has also been shown to be effective at reducing hyperglycemia and improving glucose tolerance in the STZ model [73]. There has been one report of the effectiveness of conophylline to improve glycemia in rodent models of T2DM.

### In Vivo Studies

Neonatal Wistar rats injected with STZ (single injection, 85 μg/g) and treated with conophylline (5 mg/kg/day, SQ injection) every other day (e.g., days 1, 3, 5, 7) for 7 days displayed reduced plasma glucose concentrations, improved glucose tolerance, and increased secretion of insulin in response to glucose over an 8-week period. At the end of the study period, there was also a significant increase in the number of Pdx1-positive ductal cells, insulin content, and pancreatic β-cell mass compared to diabetic rats [73].

Similarly, in STZ-exposed neonatal Wistar rats (single injection, 100 μg/g), 1 week of conophylline treatment (2 mg/kg/day, i.p. injection) improved glucose tolerance and decreased plasma glucose measured 8 weeks after STZ injection. Pancreatic insulin content and β-cell mass were also increased in the conophylline-treated group compared to the STZ-only controls, 8 weeks after STZ injection [74]. Additionally, conophylline administration (0.9 mg/kg/day, oral gavage) for 4 weeks improved glucose tolerance, increased pancreatic insulin, reduced islet fibrosis, and increased the number of MafA-positive β-cells in Goto-Kakizaki rats, a non-hypertensive model of diabetes [75].

### In Vitro Studies

In acinar carcinoma cells (AR42J), a pancreatic progenitor cell line, conophylline treatment (0.1 mg/mL for 72 h) promoted 20% of the cells to become insulin-positive, although these cells were found to be Nkx6.1-negative [76]. It is postulated that conophylline activates p38 MAPK, which in turn stimulates the production of neurogenin3, a transcription factor critical for endocrine determination of AR42J cells [72, 76]. Conophylline (0.1 μg/mL for 72 h), in the presence of nicotinamide, triggered porcine islet-like cell clusters to differentiate into β-cells. Nicotinamide plus conophylline increased the expression of insulin, Pdx1, neurogenin3, and neuroD/Beta2, all of which play a role in the development and differentiation of β-cells [77]. Conophylline also promotes insulin-producing cell formation from bone marrow mesenchymal cells when used in combination with betacellulin-delta4 [78]. Although these cells express mature β-cell transcription factors, their ability to secrete insulin under stimulatory glucose concentrations is less than two-fold [78].

In neonatal mouse islets, conophylline treatment (100 ng/mL for 72 h) increased mRNA expression of insulin and the number of Pdx1-positive cells, similar to results obtained with the use of activin A [73]. Thus, it is possible that conophylline acts similarly to activin A, a protein complex that stimulates β-cell differentiation via activation of p38 MAPK. Importantly, it has been suggested that activin A induces apoptosis, whereas it appears that the concentration of conophylline that successfully promoted differentiation of endocrine cells from AR42J cells and porcine islet-like clusters does not decrease cell viability [76, 77].

## Curcumin

Curcumin is a diarylheptanoid and a major component of *Curcuma longa*, where the spice turmeric is derived from drying out the plant and grinding it into powder (Table 1). Turmeric is commonly used in cooking and medicine in southern Asia and curcumin is the primary compound thought to provide the medicinal properties of the plant [79]. Curcumin was first isolated in 1842 but the chemical structure was not reported until 1910 [80]. Curcumin has been reported to modulate numerous signaling pathways and has been demonstrated to have anti-oxidant, anti-inflammatory, anti-proliferative, and wound healing properties. A very large body of literature exists demonstrating a beneficial role for curcumin in animal models of diseases and conditions including inflammatory disorders, e.g., intestinal inflammation, neurological disorders, gastrointestinal disorders, and cancer treatment [80]. Moreover, curcumin has been shown to exhibit beneficial properties in various animal models of obesity and T2DM [81–84], T1DM [85], and in humans with prediabetes [86] and T2DM [87].

### In Vivo Studies

In high-fructose-fed Wistar rats (60% fructose by weight), 10 weeks of curcumin supplementation (200 mg/kg/day, oral gavage) reduced FBG, fasting insulin, HOMA-IR, and inflammation as measured by circulating tumor necrosis factor-α (TNFα) and C-reactive protein (CRP), compared to chow-fed animals. Glucose intolerance seen in high-fructose-fed animals was completely ameliorated with curcumin supplementation [81]. In HFD-fed (35.9% fat) Wistar rats injected with STZ to induce diabetes (single injection, 39 mg/kg), 7 weeks of curcumin supplementation (150 or 250 mg/kg/day, oral gavage) reduced FBG, improved glucose tolerance, and improved insulin tolerance [82]. In high-fat diet-induced obese C57BL/6 J mice (35% fat) compared to chow-fed mice, 8 weeks of curcumin supplementation (approximately 60 mg/day, in diet) was effective in reducing HbA1c with a concomitant decrease in body weight and fat mass [83]. A similar decrease in HbA1c was seen in genetically obese *ob/ob* mice receiving a 4% fat diet supplemented orally with curcumin [83]. In *db/db* mice, curcumin supplementation (0.2 g/kg diet) for 6 weeks reduced FBG and improved glucose tolerance, when compared to the non-curcumin-treated *db/db* group [84]. Long-term curcumin consumption (60 mg/day in diet, for 75 days) also improved non-fasting blood glucose, HbA1c, and serum insulin in *db/db* mice [83].

In STZ-treated diabetic SD rats (65 mg/kg single i.p. injection), curcumin treatment (100 mg/kg/day, oral gavage) for 7 days reduced FBG, HbA1c, plasma protein oxidation, an indicator of oxidative stress, and the plasma inflammatory markers TNFα, interleukin-6 (IL-6), and monocyte chemoattractant protein-1 (MCP-1) compared to vehicle control diabetic rats [85].

In a clinical population of male and female prediabetic patients, treatment with curcumin (250 mg/day, oral capsule) for 9 months resulted in reduced FBG, HbA1c, HOMA-IR, and plasma C-peptide levels, and increased HOMA-β when compared to placebo. Additionally, 16% of the placebo group was diagnosed with T2DM at the end of the study, while none of the curcumin group progressed beyond prediabetes [86]. Conversely, in a population of male and female individuals with T2DM, oral curcumin supplementation (1500 mg/day; oral capsules) for 10 weeks reduced FBG, but no significant changes in serum insulin, HbA1c, HOMA-IR, or HOMA-β were observed [87].

### In Vitro Studies

In isolated mouse islets, pretreatment with curcumin (10 μM for 24 h) before STZ exposure (1 mM for 8 h) reduced NO, peroxynitrite, and MDA concentrations. Curcumin also decreased activated PARP. These scavenging effects prevented the STZ-associated reduction in cellular viability and insulin secretion [88]. In rat INS-1 cells, incubation with curcumin over a concentration response (5, 10, and 15 μM for 24 h) partially restored the loss of insulin and Pdx1 expression associated with chronic high glucose exposure (30 mM for 48 h). Curcumin also ameliorated the chronic glucose-dependent attenuation of glucose-stimulated insulin secretion (GSIS). However, higher concentrations of curcumin (20, 40, and 60 μM for 24 h) decreased cell viability [89], indicating a narrow range of therapeutic potential.

A myriad of possible molecular targets have been identified for curcumin. For example, curcumin downregulates phosphodiesterase (PDE) expression and activity in MIN6 cells and a pancreatic β-cell line (HP62), influencing the enzymes that catalyze the breakdown of cAMP, which leads to the increased intracellular concentration of cAMP and consequently an enhancement of insulin secretion at stimulatory glucose concentrations [90]. Additional targets of curcumin include the protein kinases JAK, MAPK, and IκKα, reducing inflammatory signaling cascades [91, 92].

#### Epigallocatechin-3-gallate

Epigallocatechin-3-gallate (EGCG, Table 1) is a polyphenolic bioactive compound found in green tea (*Camellia sinensis*) and is purported to have beneficial effects for a variety of diseases including obesity, cancer, cardiovascular diseases, inflammatory diseases [93], and glutamate neurotoxicity [94]. EGCG has been shown to reduce plasma glucose concentrations and improve glucose tolerance in numerous models, including obesity and T2DM [95], non-obese T2DM [96], T1DM [97], and in STZ-treated rodents [98]. A number of in vitro studies using insulinoma cell lines and isolated islets have shown that EGCG mediates a direct anti-inflammatory effect on the β-cell [97–100].

### In Vivo Studies

In *db/db* mice, 10 weeks of EGCG supplementation (1% w/w of diet) reduced FBG. A greater decrease in FBG was seen with dietary supplementation of rosiglitazone, as early as 5 weeks into the regimen; however, EGCG did not promote an increase in body weight as was seen in rosiglitazone-treated *db/db* mice. Both EGCG and rosiglitazone improved glucose tolerance in *db/db* mice after 10 weeks of treatment correlating with increased insulin output during OGTT. Moreover, EGCG and rosiglitazone promoted an increase in pancreatic islet number compared to *db/db* control animals. However, an improvement in insulin sensitivity was only seen in the rosiglitazone-treated mice [95]. Male Goto-Kakizaki rats, a non-obese model of T2DM, treated with EGCG (100 mg/kg/day, oral gavage) for 3 months also displayed improved FBG and glucose tolerance compared to non-supplemented GK rats [96].

In female NOD mice, EGCG supplementation (0.05% of drinking water) beginning at 5 weeks of age for 17 weeks decreased diabetes onset by more than 40% with a significant improvement in non-fasting blood glucose levels. This correlated with a dramatic increase in the survival rate of NOD mice at 30 weeks of age. EGCG supplementation also improved glucose tolerance, lowered HbA1c, and increased plasma insulin concentration. These physiological alterations occurred despite no change in pancreatic islet insulitis during EGCG treatment [97].

In STZ-injected C57BL/KsJ mice (40 mg/kg, 5 days, i.p. injection), supplementation of EGCG for 10 days both during and after STZ administration (100 mg/kg/day, i.p. injection) reduced STZ-induced hyperglycemia and promoted an increase in islet size relative to STZ-treated controls, as assessed by hematoxylin/eosin staining of fixed pancreatic tissue [98].

### In Vitro Studies

In RINm5F cells, EGCG treatment (20–200 μg/mL for 24 h in the presence of cytokines) protected against interferon-γ (IFNγ) and interleukin-1β (IL-1β)-mediated cell death. Additionally, EGCG treatment inhibited cytokine-induced translocation of NF-κB to the nucleus, iNOS expression, and NO production [99]. When RINm5F cells were exposed to a combination of inflammatory cytokines IL-1β, TNFα, and IFNγ, EGCG pretreatment (40 μM for 24 h) reduced both iNOS abundance and NO production, prevented β-cell death, and partially reversed the cytokine-dependent decrease in glucose-stimulated insulin secretion [100].

In islets isolated from C57BL/KsJ mice, EGCG treatment (20, 50, 100, and 200 μg/mL for 24 h in the presence of inflammatory cytokines (IL-β and IFNγ) improved cell viability in a dose-dependent manner when compared to control cytokine-treated islets [98].

EGCG pretreatment (1 and 10 μM for 12 h) of human islets before exposure to inflammatory cytokines (IL-1β and IFNγ for 48 h) dose-dependently increased cell viability and decreased caspase-3 activity compared to control (cytokine only treated) islets [97].

## Genistein

Genistein is an isoflavone found in several plants, with the most notable being soybeans [101, 102] (Table 1). Genistein was first isolated in 1899 from *Genista tinctoria* from where the name genistein is derived [103]. Genistein has been reported to have cardioprotective effects, improve menopause symptoms, reduced incidence of some cancers, and have anti-depressant abilities [104]. Genistein has been studied in a number of rodent models of diabetes (T1DM, T2DM, and β-cell death by STZ) and has shown consistent glucose-lowering abilities across model systems [105–108]. Beneficial outcomes on plasma glucose and insulin sensitivity have also been observed in a human study of non-diabetic individuals [109]. These anti-diabetic properties of genistein may be due to its direct action on the β-cell, as shown in several in vitro studies using insulinoma cell lines and isolated islets [106, 110–114]. However, genistein may only provide beneficial effects below a certain concentration threshold [111].

### In Vivo Studies

In STZ-injected C57BL/6 mice (45 mg/kg, 5 days), genistein treatment (10 mg/kg, 3 days/week, i.p. injection) for 10 weeks modestly, yet significantly, reduced FBG. Despite a detectable reduction in blood glucose, no improvement was seen in the number of islets or plasma insulin concentration, in genistein-treated diabetic animals compared to untreated diabetic mice [105]. Administration of genistein at a greater dose (250 mg/kg in diet) for 4 weeks in STZ-injected C57BL/6J mice (40 mg/kg, 5 days) improved glycemic control, measured as non-fasting blood glucose and glucose tolerance, and was observed relative to STZ-injected mice on the control diet. Genistein restored both plasma insulin levels and β-cell mass to values similar to the control animals that did not receive any STZ. Furthermore, dietary genistein supplementation partially counteracted the STZ-associated β-cell loss by stimulating proliferation (measured by BrdU positivity) and reducing TUNEL-positive β-cells [106].

To generate a model of non-genetic obesity and T2DM, C57BL/6 mice were fed a high-fat diet (60% fat) for 4 weeks followed by a single injection of STZ (90 mg/kg). Supplementation of the HFD with genistein (250 mg/kg/day) improved FBG levels compared to obese, T2DM (HFD + STZ) mice receiving no genistein; however, glucose levels were still significantly elevated relative to high-fat or chow-fed animals that did not receive an injection of STZ. Compared to the chow diet alone (no STZ), plasma insulin levels were elevated in the HFD group (no STZ). As expected in HFD+STZ mice, circulating levels of insulin were far lower than those in chow mice, yet dietary administration of genistein restored plasma insulin to levels in HFD+STZ mice. Islet β-cell mass tended to correlate with circulating insulin levels in this study. Furthermore, genistein reduced STZ-induced β-cell apoptosis assessed by staining pancreatic sections for active caspase-3 [107].

Beginning at 9 weeks of age, dietary supplementation with genistein (0.2 g/kg of diet) significantly improved fasting plasma glucose levels compared to untreated NOD mice. This intervention also completely prevented the onset of hyperglycemia in female NOD mice compared to control animals. Further, genistein supplementation significantly decreased serum FFA and TG levels. In agreement with an increase in circulating levels of insulin and C-peptide, the number of insulin-positive cells, assessed by immunohistochemistry of pancreatic sections, was also increased following 9 weeks of genistein supplementation [108]. Moreover, in human studies, 2 years of genistein administration (54 mg/day) reduced FBG and insulin levels, and improved insulin sensitivity as assessed by HOMA-IR, when compared to the placebo in non-diabetic post-menopausal women [109].

### In Vitro Studies

Insulinotropic effects of genistein appear to vary depending on the concentration used and the length of incubation time. For example, acute treatment of isolated mouse islets and the pancreatic β-cell line INS-1 with lower genistein concentrations (5 μM for 30 m) potentiated GSIS [110], and moderate concentrations (50 μM for 1 h) also increased GSIS in isolated pancreatic mouse islets [111]. Conversely, high concentrations of genistein (200–500 μM for 1 h) dose-dependently inhibited GSIS in isolated rat islets [111]. In a time-dependent study, 100 μM genistein was shown to increase insulin secretion in isolated rat islets at 24 and 48 h [112]; however, the observed fold increase gradually decreased at 3 and 4 days of exposure to genistein.

Low concentrations of genistein induced proliferation, measured via BrdU incorporation, in both INS-1 cells (0.1–10 μM for 24 h) and human islets (1 and 5 μM for 24 h) [106]. Alternatively, exposure to greater concentrations of genistein (100 μM for 24 h) increased β-cell apoptosis in RINm5F at 4 h [113] and 24 h [114], and also following 24h treatment in rat and human islets [114]. The observed increase in apoptosis associated with high genistein concentrations may be through inhibition of topoisomerase-II [113], as has been reported in mouse lymphoma cells [115]. Topoisomerase-II inhibition can lead to DNA fragmentation, apoptosis, and induce G2/M cell cycle arrest [116].

Increases in intracellular cAMP and subsequent activation of protein kinase A (PKA) were shown to be responsible for both the proliferative effects of genistein [106] and the insulin-secreting activity of pancreatic β-cell lines and mouse islets [110]. Genistein also induced the ERK1/2 signaling pathway [106]. Genistein has anti-oxidant and anti-inflammatory properties. These effects are achieved by scavenging free radicals, decreasing lipid peroxidation, and inhibiting the expression of COX and myeloperoxidase (MPO) [117, 118]; however, this anti-oxidant defense mechanism has not been tested in the context of β-cell function.

## Gingerol

First isolated in 1879, gingerol is the predominant and most important phenol found in the ginger plant (*Zingiber officinale*), a plant widely used as both a spice and traditional medicine [119] (Table 2). Ginger has been reported to have anti-inflammatory, cardioprotective, anti-nausea, and neuroprotective effects [120]. Furthermore, gingerol has exhibited anti-diabetic properties including an improvement in FBG, increased glucose tolerance, improved insulin secretion, and reduced β-cell death in multiple models of diabetic rodents [121–123]. However, it is important to note that these effects are not consistent from study-to-study, which could be due to the dose of gingerol used or the route of administration [121, 122]. Moreover, whether gingerol can mediate beneficial effects in rodent models and human subjects with T1DM or individuals with obesity and diabetes has not yet been determined.

### In Vivo Studies

Four weeks of gingerol supplementation (0.05% in the diet) in *db/db* mice improved FBG concentrations compared to *db/db* mice fed a control diet but did not return the blood glucose to levels observed in non-diabetic control mice. Gingerol supplementation also improved insulin sensitivity, blood lipid profile, and reduced serum TNFα concentration compared to untreated *db/db* mice [121].

In a separate study using *db/db* mice, gingerol administration for 4 weeks (200 mg/kg/day, oral gavage) was shown to be as effective as glibenclamide in improving glucose tolerance compared to vehicle control mice. Improved glucose tolerance was accompanied by a significant increase in insulin secretion in both gingerol- and glibenclamide-treated mice during the OGTT. However, unlike glibenclamide treatment, which lowered FBG levels as early as 1 week into the 4-week treatment period, gingerol administration showed no glucose-lowering effects or alterations in blood insulin levels in either the fed or fasted state compared to untreated vehicle controls. Interestingly, following a glucose bolus, the gingerol-treated animals displayed increased plasma GLP-1 and reduced plasma dipeptidyl peptidase-4 (DPP4) concentrations and activity. The authors conclude that increased GLP-1 in response to glucose in gingerol-treated mice “mediates the glucose-induced insulin secretion activity” from pancreatic β-cells, as this effect is blocked by the addition of the PKA inhibitor H89 [122]. Furthermore, in islets from *db/db* mice, gingerol was shown to enhance mRNA expression and abundance of two factors involved in insulin vesicle docking, *Rab27* and *Slp4-a*, suggesting that gingerol may play a role in exocytosis of insulin-containing vesicles [122].

In Swiss albino mice made diabetic by arsenic exposure (3 mg/kg/day, oral gavage, 12 weeks), gingerol treatment for 3 weeks (50 and 75 mg/day, oral gavage) dose-dependently reduced pancreatic arsenic deposition and arsenic-induced ROS accumulation. Both concentrations of gingerol reduced arsenic-induced hyperglycemia and improved both FBG levels and glucose tolerance. Furthermore, treatment with gingerol significantly improved plasma insulin concentrations compared to arsenic-induced diabetic control animals [123].

### In Vitro Studies

In RIN-5F cells, gingerol treatment (25–100 μM for 4 h) dose-dependently reduced basal ROS concentrations as measured by DCFHA-DA. Gingerol (50 μM for 4 h) also reduced ROS activity generated by the addition of artificial advanced glycation end-product (AGE)2 [121]. At present, there are limited in vitro studies published in English language journals. Clearly, more research is needed in this important area to determine how gingerol mediates its anti-diabetic effects.

## Gymnemic Acid

Gymnemic acid is a class of chemical compounds isolated from the leaves of the plant *Gymnema sylvestre*, also known as the Australian cow plant (Table 2). Gymnemic acid was first isolated in 1888. These compounds interact with taste receptors on the tongue to temporarily suppress the taste of sweetness [124]. *Gymnema sylvestre* is also known to have anti-oxidant, anti-inflammatory, antibiotic, anti-viral, hepatoprotective, anti-cancer, and lipid-lowering activities [125]. Gymnemic acid is considered to be the primary compound responsible for the anti-diabetic effects of *Gymnema sylvestre* [126]. Gymnemic acid has been extensively studied in models of hyperglycemia including chemical induction models (STZ [127] and alloxan [128]), *db/db* mice [129], and STZ in combination with HFD [130]. The most common outcome across these various model systems is the ability of gymnemic acid, regardless of route of administration, to reduce plasma glucose levels. These glucose-lowering effects may be modulated by increased insulin secretion, as seen in in vitro experiments using insulinoma cell lines and islets [131, 132].

### In Vivo Studies

A single dose of gymnemic acid (13.4 mg/kg, i.p. injection) significantly lowered plasma glucose levels 6 h after administration in STZ-induced diabetic ddY mice (150 mg/kg, single injection). By comparison, a single dose of glibenclamide significantly lowered glucose levels by 4 h and remained lower by 6 h compared to the STZ control group. Gymnemic acid administration to STZ mice also significantly elevated plasma insulin levels compared to STZ controls [127]. In alloxan-induced diabetic rats (100 mg/kg, single injection), 30 days of gymnemic acid treatment (200 mg/kg/day, oral gavage) helped to restore alloxan-induced β-cell degeneration as determined by histopathological examination of pancreatic sections [128].

In *db/db* mice, 8 weeks of gymnemic acid treatment (100 mg/kg/day, oral gavage) reduced FBG and HbA1c, improved blood lipid profiles, restored glucose tolerance, and improved inflammation as measured by reduced circulating levels of TNFα, MCP-1, and IL-6. Gymnemic acid treatment increased serum insulin with a concomitant increase in insulin/glucagon ratios detected by immunofluorescence staining of pancreatic tissue. Gymnemic acid also promoted β-cell proliferation as assessed by Ki67 staining of pancreatic sections [129].

In SD rats rendered diabetic through a combination of high-fat diet feeding (32% fat and 44% sugar, estimated based on the table provided) and STZ injection (30 mg/kg, single injection), gymnemic acid treatment for 6 weeks (40 and 80 mg/kg/day, oral gavage) reduced FBG, glucose intolerance, insulin resistance, and glycated serum protein levels compared to T2DM rats. A reduction in fasting serum insulin compared to T2DM animals was seen only in high-dose gymnemic acid. However, HOMA-β calculations show a dose-dependent effect [130].

### In Vitro Studies

In several β-cell lines (HIT-15, RINm5F, and MIN6), treatment with an extract of *Gymnema sylvestre* (termed GS4), which contains gymnemic acid, at 0.125, 0.25, and 0.5 mg/mL for 1 h increased basal insulin secretion in a dose-dependent manner [133]. However, the ability of GS4 to trigger insulin release is the result of increased membrane permeability. The high saponin glycoside content found in gymnemic acid increases plasma membrane permeability resulting in insulin leakage from the cell rather than regulated exocytosis [133].

In MIN6 cells, treatment with an aqueous extract of *Gymnema sylvestre* leaves termed OSA increased basal- and potentiated glucose-stimulated insulin secretion [131, 132]. Unlike the GS4 preparation [133], OSA did not promote increased trypan blue staining in human islets [132]. Insulin release was most likely due to the increase in OSA-dependent intracellular calcium concentration; accordingly, the calcium channel inhibitor nifedipine abolished this response. A similar increase in basal and potentiation of glucose-stimulated insulin secretion was seen in human islets treated with OSA [132]. In a separate study, MIN6 cells exposed to gymnemic acid (5 μg/mL for 36 h) decreased high glucose-induced caspase-3 activity and apoptosis and induced autophagy under both normal and high glucose stress by inhibiting the activity of mTOR1 [134]. In isolated islets from ICR mice, treatment with OSA (0.25 mg/mL for 30 min) substantially increased basal insulin secretion; this response was partially attenuated by nifedipine [131].

## Kinsenoside

Kinsenoside is a key active component isolated from the genus *Anoectochilus*, such as *A. roxburghii*, also known as the jewel orchid (Table 2). Kinsenoside has been shown to have great therapeutic potential with vascular protective, anti-inflammation, and anti-hyperliposis effects among some of its myriad properties [135]. This medicinal plant has been used for centuries in traditional Chinese medicine in the treatment of numerous ailments; however, the active compound was not isolated until 1993 [136]. Surprisingly, literature is quite limited regarding kinsenoside and diabetes. To date, the only study performed using kinsenoside is in the STZ model of acute inflammation and hyperglycemia [137], with no reports of its effects in rodent models of T1DM or T2DM, or in patients with diabetes. In addition, no in vitro studies using cell lines or isolated islets have been performed to test for direct effects of kinsenoside on the β-cell.

### In Vivo Studies

In STZ-induced diabetic Wistar rats (50 mg/kg, single injection), kinsenoside treatment (15 mg/kg/day) for 21 days improved glucose tolerance measured via OGTT. Glucose tolerance was also improved in non-diabetic animals. In a side-by-side analysis with metformin, both metformin and the highest dose of kinsenoside restored plasma insulin levels in STZ-treated rats to those of non-diabetic controls. Histopathological examination of the pancreas showed that unlike metformin, treatment with kinsenoside appears to promote a dose-dependent alleviation of STZ-induced loss in insulin-positive cells. However, no quantification of the insulin-positive area or β-cell mass were shown in this report, and the representative pancreatic section shows a single islet for each treatment group [137].

Kinsenoside treatment in STZ-induced diabetic rats increased the activity of both total superoxide dismutase and scavenging hydroxyl radical in serum to a similar extent as metformin. Although the serum NO content was reduced by kinsenoside, no change was seen in NOS or iNOS activity [137].

## Nymphayol

Nymphayol is a sterol present in *Nymphaea caerulea* also known as the blue lotus (Table 2). Nymphayol containing plants are widely used in Ayurveda and Siddha medicine for the treatment of diabetes, inflammation, liver disorders, and many other ailments [138]. Nymphayol has been shown to have anti-diabetic properties in rats rendered diabetic using the drug STZ [139–141]. These effects may be mediated, at least in part, through its ability to increase insulin secretion, as demonstrated in an insulinoma cell line [140].

### In Vivo Studies

Using Wistar rats made diabetic by STZ (40 mg/kg, single injection), an oral gavage of nymphayol (50 mg/kg/day, for 25 days) was shown to be as effective as the FDA-approved drug pioglitazone in reducing glucose intolerance and improving serum lipid profiles [139]. In addition, nymphayol administration (20 mg/kg/day, oral gavage) for 45 days, given 7 days after onset of diabetes in STZ-injected Wistar rats (45 mg/kg, single injection), was able to reduce fasting plasma glucose and HbA1c compared to diabetic rats. Moreover, the effect of nymphayol was similar to animals administered glibenclamide. Likewise, nymphayol administration was as successful as glibenclamide at restoring plasma insulin concentrations that were depleted by STZ exposure [140, 141].

### In Vitro Studies

In RIN5 cells, nymphayol treatment (20 or 40 μM for 3 h) dose-dependently increased insulin secretion when compared to the control; this was observed under both low glucose (5.5 mM) and high glucose (25 mM) concentrations [140]. As a possible mechanism for these effects, nymphayol may increase the expression and/or activity of PPARγ which has been shown in adipose tissue [139]. Although no studies to date have tested the ability of nymphayol to regulate PPARγ abundance or activity in the β-cell, enhancing PPARγ activity in the insulin-producing cell leads to increased intracellular Ca^2+^ concentration and consequently increased insulin secretion [142]. Further studies are required to directly test this possibility.

## Paeoniflorin

Paeoniflorin, first isolated in 1963, is a glycoside and major component of *Paeonia lactiflora* [143] (Table 2). Paeoniflorin has been demonstrated to have anti-inflammatory and immune regulatory effects beneficial for several autoimmune diseases, such as rheumatoid arthritis, allergic contact dermatitis (ACD), psoriasis, and ulcerative colitis. This molecule is also reported to have cardioprotective, neuroprotective, and hepatoprotective effects [144]. Paeoniflorin has shown promising results in models of hyperglycemia [145] and insulin resistance [146]; however, it remains to be determined whether paeoniflorin can improve diabetic parameters in rodents and humans with T1DM and T2DM.

### In Vivo Studies

In Wistar rats exposed to STZ (60 mg/kg, single injection), a single i.v. injection of paeoniflorin (1, 5, or 10 mg/kg) reduced plasma glucose in a dose-dependent manner when compared to the non-treatment diabetic group. The non-diabetic Wistar rats also experienced reduced plasma glucose, but no dose-dependent effects were observed [145]. In Sprague Dawley rats given a 20% fructose drink, which provides a model of hyperinsulinemia and insulin resistance, but not hyperglycemia, 8 weeks of paeoniflorin treatment (10, 20, 40 mg/kg/day, oral gavage) improved glucose tolerance to a similar extent as the insulin sensitizer pioglitazone. In fructose-fed rats, paeoniflorin decreased serum levels of TG, insulin, and glucagon. All three doses of paeoniflorin improved insulin sensitivity, represented by the HOMA-IR index; however, in this study, pioglitazone was the superior insulin-sensitizing intervention. Similar improvements in these biochemical markers were also seen with pioglitazone treatment [146].

### In Vitro Studies

In INS-1 cells, paeoniflorin pretreatment (20, 40, 80 μM for 2 h) before STZ exposure (3 mM for 24 h) dose-dependently restored the STZ-induced loss in insulin content, SOD activity, and BCL-2 expression. Paeoniflorin treatment also reduced STZ-induced caspase-3 activity, Bax expression, ROS production, and MDA concentrations, as well as suppressed STZ-induced activation of p38 MAPK and JNK pathways, all in a dose-dependent manner. The ability of paeoniflorin to inhibit these pathways promoted β-cell survival [147].

## Quercetin

Quercetin is a natural polyphenolic pigment flavonoid found in a wide variety of plants, including fruits and vegetables such as buckwheat, apples, berries, and red onions (Table 3). The therapeutic potential of quercetin has been continually investigated in many distinct experimental paradigms. It has been reported to have anti-oxidant, anti-diabetic, anti-tumor, anti-microbial, anti-inflammatory, immune-regulatory, and cardioprotective properties [148]. Quercetin has been extensively studied in rodent models of diabetes, including STZ-induced diabetes in Wistar rats and genetically obese *db/db* mice. Regardless of the route of administration, quercetin has been shown to have a glucose-lowering ability that is often, but not always, accompanied by an alteration in circulating insulin levels [149–153]. In vitro studies using insulinoma cell lines and isolated islets reveal that quercetin can enhance insulin secretion as well as provide anti-inflammatory and anti-oxidant actions directly in the β-cell [154–160]. Furthermore, quercetin is or has been investigated in phase II clinical trials for the treatment of T2DM (NCT00065676 and NCT01839344).

### In Vivo Studies

In Wistar rats exposed to STZ (50 mg/kg, single injection), 4 weeks of quercetin supplementation (15 mg/kg/day, i.p.), 3 days prior to STZ injection, showed significant anti-hyperglycemic effects relative to STZ controls. Circulating insulin were partially restored after quercetin supplementation in STZ-exposed rats. This increase in serum insulin was accompanied by a ten-fold increase in immunoreactive insulin in islet β-cells, yet remained 50% lower than control animals. A decrease in pancreatic abundance of MDA as well as an increase in pancreatic SOD, GSH-Px, and CAT accompanied these metabolic changes [149].

In a study from a separate group, quercetin administration (25 mg/kg/day, i.p.) for 33 days beginning 3 days prior to STZ induction in Wistar rats (75 mg/kg, single injection) prevented the onset of hyperglycemia and restored plasma insulin concentrations to control values. Quercetin pretreatment reduced losses in β-cell mass, shown via histological examination of pancreatic tissue. Quercetin administration to STZ-exposed rats also reduced serum NO and improved anti-oxidant defense as measured by increased abundance of pancreatic CAT, SOD, GSH-Px, and a reduction in MDA concentration [150].

In Wistar rats exposed to STZ (50 mg/kg, single injection) treatment with the glycosidic form of quercetin known as rutin (25, 50, and 100 mg/kg/day, oral gavage) for 45 days dose-dependently decreased plasma concentrations of glucose [151]. Using the highest dose from this study, these authors confirmed this anti-hyperglycemic effect and further showed that rutin increased plasma insulin levels compared to diabetic controls. Rutin administration in STZ rats increased pancreatic concentrations of GSH, SOD, catalase, and GSH-Px [152].

In STZ-exposed Sprague Dawley rats (50 mg/kg, single injection), administration of quercetin-containing (100 mg/kg) soluble starch via gastric intubation following an overnight fast significantly reduced post-prandial hyperglycemia compared to control rats administered starch only [153]. In a hypertriglyceridemia HFD-fed model (20% lard and 3% cholesterol) SD rats that underwent acute pancreatitis induction, quercetin (100, 150, 200 mg/kg, i.p. injection; 30, 60, 120, 180 min following acute pancreatitis induction) dose-dependently reduced pancreatic damage and suppressed pancreatic inflammation by reducing mRNA expression of inflammatory markers including NF-κB, IL-1β, IL-6, and TNFα [161].

In *db/db* mice, quercetin supplementation (0.08% diet) for 7 weeks lowered fasting glucose concentrations and HbA1c without altering plasma insulin levels [153].

### In Vitro Studies

In INS-1 cells, 1 h of quercetin treatment (20 μM) increased insulin secretion 1.5-fold under non-stimulated conditions, and when the cells were exposed to glucose (8.3 mM) or other secretagogues (glibenclamide), quercetin potentiated insulin secretion [154]. Quercetin had no effect on basal insulin secretion, but potentiated glucose-stimulated insulin secretion in isolated Wistar rat islets [154]. Similarly, INS-1 cells treated with quercetin (20 μM for 1 h) more than doubled basal insulin secretion when compared to the non-treated control. In the same study, isolated rat islets treated with quercetin (10 and 20 μM for 30 m) experienced a dose-dependent increase in insulin secretion [155]. Both basal and glucose-stimulated insulin secretion were enhanced 1.7- and 2.7-fold, respectively, in the presence of 50 μM quercetin in INS-1 cells [156]. Quercetin also potentiated GSIS in INS-1E cells. This was associated with a corresponding increase in the expression of Glut2, Gck, and Ins1 at stimulatory glucose conditions [157].

The ERK1/2 signaling pathway also plays a role in the regulation of glucose-stimulated insulin secretion in vitro [162]. Interestingly, quercetin, in the presence of stimulatory concentrations of glucose, robustly induces phosphorylation of ERK1/2; a modest activation of ERK was seen in the presence of glucose or quercetin alone. This effect was decreased in the presence of the ERK inhibitor U0126, but was independent of PKA. Given that quercetin potentiated the KCl-induced increase in intracellular Ca^2+^, it was postulated that quercetin activates ERK1/2 through increased intracellular calcium, which sensitizes β-cells to insulin secretagogues and thereby potentiates insulin secretion [154].

Increased insulin release stimulated by quercetin is thought to be mediated through transient inward rectifying K_ATP_ channel inhibition and stimulation of voltage-gated Ca^2+^ channels. It is unclear how quercetin inhibits inward rectifying K_ATP_ channels. However, in β-cells, quercetin can directly activate L-type Ca^2+^ channels without altering membrane depolarization, leading to increased intracellular calcium (Ca^2+^_i_) which positively influences insulin secretion [155, 156].

Treatment with quercetin protected INS-1 cells against H_2_O_2_-induced cell death and attenuated the H_2_O_2_-associated insulin secretion loss [154]. In RINm5F cells exposed to IL-1β, quercetin pretreatment (10 μM for 1 h) reduced nitrite production and iNOS expression, as well as inhibited IκBα phosphorylation. The reduction in insulin secretion observed by overnight exposure to IL-1β was also mitigated [158]. Similarly, a concentration response of quercetin (5, 10, and 20 μM for 2 h) tested in RINm5F cells partially restored the cytokine-mediated decrease in GSIS (IL-1β, TNFα, IFNγ for 24 h) [159]. Quercetin pretreatment protected against cytokine-induced loss in viability and reduced cytokine-mediated ROS and NO production. The higher dose of quercetin (20 μM) also inhibited the loss of IκBα and ameliorated the cytokine-associated increase in iNOS, caspase activity, NF-κB nuclear expression, and Bax expression [159].

Co-treatment of RINm5 cells in the presence of 25 and 50 μM quercetin, and the cytokines IL-1β and IFNγ, partially protected against cytokine-induced losses in viability. Quercetin co-treatment also decreased cytokine-dependent production of nitrite and iNOS expression, translocation of NF-κB to the nucleus, and IκBα phosphorylation and degradation. Cytokine-mediated impairment in insulin secretion in rat islets was also prevented by treatment with quercetin [160].

Long-term application of high-dose quercetin (50 μM for 48 h) in INS-1 cells can produce negative outcomes, such as inhibiting cell proliferation and promoting apoptosis; this is likely via inhibition of PI3K/Akt signaling [156].

## Resveratrol

Resveratrol is a stilbenoid polyphenol produced by plants in response to injury (Table 3). This polyphenol, first isolated in 1939, is found in many berries as well as the skin of grapes [163]. In clinical trials, resveratrol has been demonstrated to improve cancer, Alzheimer’s disease, cardiovascular disorders, and non-alcoholic fatty liver disorder [164]. Anti-diabetic effects of resveratrol (lowering of FBG, elevated GSIS, and improvements in circulating insulin levels) have been noted in rodent models of hyperglycemia, glucose intolerance, and T2DM [165–167]. Beneficial outcomes of resveratrol supplementation have been noted in rhesus monkeys [168], as well as in humans with T2DM [169, 170]. Resveratrol enhances insulin secretion in vitro with effects in cultured β-cell lines and isolated islets [90, 171, 172]. Furthermore, resveratrol has been shown to have potent anti-inflammatory properties in the β-cell [173]. In addition, resveratrol is or has been used in various clinical trials for the treatment of obesity, insulin resistance, and/or T2DM (NCT01677611, NCT01158417, NCT02216552, and NCT01354977).

### In Vivo Studies

In STZ-exposed Wistar rats (50 mg/kg, single injection) also given nicotinamide (110 mg/kg, single injection), 30 days of resveratrol supplementation (5 mg/kg/day, oral aqueous solution) lowered FBG and HbA1c. Rats receiving resveratrol demonstrated greater plasma insulin concentrations compared to the diabetic control and insulin levels were similar when compared with the oral hypoglycemic agent gliclazide. Resveratrol supplementation decreased plasma levels of NO and inflammatory cytokines (TNFα, IL-1 β, and IL-6). In pancreatic tissue, resveratrol increased the activity of a number of enzyme anti-oxidants including SOD, catalase, GPx, and GST compared to diabetic control rats. Improved islet architecture and β-cell survival was also observed via TEM [165].

In high-fat-fed C57BL/6 J mice (59% fat), resveratrol supplementation (400 mg/kg/day) for 16 weeks reduced FBG and plasma insulin. Resveratrol intake improved glucose tolerance beyond even the chow control. Additionally, resveratrol improved glucose-stimulated insulin secretion and decreased pancreatic TG content relative to the high-fat-fed animals. Decreased markers of apoptosis were also present in resveratrol-treated animals [166].

Following 8 weeks of high-fat high-sucrose feeding (60% common chow, 10% lard, 10% egg yolk powder, and 20% sucrose), SD rats were injected with a single dose of STZ (40 mg/kg) to promote diabetes onset. Diabetic rats were administered a single dose of resveratrol (30 mg/kg, i.g.) and monitored for an additional 8 weeks. Significant improvements in post-prandial glucose concentration were seen by 4 weeks compared to diabetic animals and maintained through the rest of the 8-week study. A substantial lowering in FBG was also observed at the end of the 8-week period. Although the single injection of resveratrol did not completely mitigate the development of diabetes, it slowed the progression of diabetes, though post-prandial glucose levels remained twice as high as non-diabetic controls. Modest improvements in circulating insulin levels in both the fasting and post-prandial states were also observed in the resveratrol-treated mice when compared to diabetic controls [167].

Intraperitoneal injection of resveratrol lowered plasma glucose and increased insulin in normoglycemic Wistar rats; however, no glucose-lowering effect was observed in STZ-induced diabetes [172]. Similarly, in adult male rhesus monkeys fed a high-fat high-sucrose diet (42% fat, 27% sucrose), 2 years of resveratrol supplementation (80 mg/day for 12 months, then 240 mg/day for 12 months) did not improve fasting glucose or insulin levels compared to high-fat high-sucrose (HFHS) control monkeys. Despite this observation, resveratrol supplementation increased insulin positive β-cell mass and decreased the α-cell/β-cell ratio compared to the HFHS group, without an overall change in islet size. The authors suggest that the alteration in the α-cell/β-cell ratio is due to the de-differentiation of β-cells into α-cells, as no changes in apoptosis or proliferation were detected. Expression of a number of essential β-cell transcription factors were decreased in the islet of HFHS monkeys including Pdx1, Nkx6.1, Nkx2.2, and Foxo1. Resveratrol protected against this deleterious phenotype. These changes were accompanied with increased insulin secretion in resveratrol-treated animals relative to HFHS controls [168].

In human studies, resveratrol has shown beneficial effects. Treatment of male and female patients with T2DM with resveratrol (250 mg/day, oral capsule) for 3 months in combination with their current anti-hypoglycemic regimen (metformin and/or glibenclamide) experienced reduced FBG and HbA1c compared to the placebo group [169]. When male and female patients with T2DM were given resveratrol (1 g/day, oral capsule) in conjunction with their current medication (oral hypoglycemic agent and/or insulin) for 45 days, FBG, HbA1c, fasting serum insulin, and insulin resistance were all significantly reduced compared to the placebo group [170].

### In Vitro Studies

In INS-1E cells, treatment with resveratrol (1, 5, and 25 μM for 24 h) dose-dependently potentiated GSIS but did not alter basal insulin secretion or insulin content. The highest dose of resveratrol (25 μM) tested also increased glucose-stimulated ATP production and O_2_ consumption in INS-1E cells [171]. When three different β-cell lines (MIN6, HIT-T15, and RIN-m5F) were treated with resveratrol at varying concentrations (3, 10, 30, and 100 μM for 1 h), the moderate resveratrol concentrations (10 and 30 μM for 1 h) increased GSIS in all cell lines. In MIN6 cells, resveratrol (10 and 30 μM) increased GSIS; however, 100 μM showed no effect. Unlike MIN6 cells, in HIT-T15 cells, resveratrol potentiated GSIS at the three highest concentrations tested. RIN-m5F cells demonstrated potentiated GSIS by resveratrol at all concentrations tested [172].

In a separate study, resveratrol treatment (0.1, 1, 10 μM for 2 h) in MIN6 cells potentiated GSIS at all doses tested, while non-stimulated insulin secretion only increased at the lower concentrations (0.1 and 1 μM), but not the higher concentration (10 μM) tested [90]. Conversely, preincubation of INS-1 cells with varying doses of resveratrol (0.2, 2, 20, and 200 μM for 1 h) did not potentiate GSIS and only a modest increase in basal insulin secretion was observed at the highest dose tested [154]. In human islets treated with the same concentrations of resveratrol (0.1, 1, and 10 μM for 2 h), both basal and glucose-stimulated insulin secretion were increased at 1 and 10 μM [90]. When human islets were treated with higher concentrations of resveratrol (25 μM for 24 h) and for longer exposures, GSIS more than doubled, but basal insulin secretion was not altered [171]. It is noteworthy that resveratrol increased GLUT2, GK, Pdx1 (aka IPF-1), TFAM, and HNF-1a expression in human islets.

In RIN-m5F cells, resveratrol pretreatment (50 μM for 3 h) effectively inhibited pro-inflammatory cytokine (IL-1β and IFNγ for 24 h)-induced activation of the NF-κB pathway as measured by iNOS expression and NO production. In addition, NF-κB transcriptional activity and acetylation of p65 at K310 were all decreased in the presence of resveratrol. Furthermore, in isolated islets from SD rats, pretreatment with resveratrol restored the cytokine-mediated decrease in GSIS and islet viability. These results were consistent with a resveratrol-dependent decrease in NF-κB pathway activation [173].

Resveratrol has been proposed to alter β-cell function in multiple ways. For example, the inhibition of both K_ATP_ and K_V_ channels in MIN6 cells, resulting in membrane depolarization, helps to explain enhanced insulin secretion [172]. Resveratrol also directly decreases the expression of PDE genes and inhibits PDE activity in MIN6 β-cells. Consequently, a concomitant increase in intracellular cAMP levels was observed [90].

Resveratrol is a known activator of Sirtuin 1 (SIRT1) [174], and the SIRT1 pathway is assumed to be a major target of resveratrol in the β-cell [171]. Resveratrol activates SIRT1, upregulating key transcription factors, which in turn promotes the expression of glucose transporter 2 (GLUT2) and various metabolic enzymes, ultimately increasing stimulus-secretion coupling. Activation of SIRT1 also upregulates the expression of Pdx1 and hepatocyte nuclear factor 1 homeobox A (HNF-1α), both of which are molecular targets linked with GLUT2 expression. Moreover, SIRT1 positively regulates glucose-stimulated insulin secretion in β-cells via downregulation of uncoupling protein 2 (UCP2) and increased ATP production [171].

The activation of SIRT1 by resveratrol also interferes with the NF-κB signaling pathway through the deacetylation of p65, resulting in inhibited iNOS expression, anti-inflammatory function, and protection from cytokine-induced β-cell death and loss of GSIS [173].

## Silymarin

Silymarin (Table 3), first isolated in 1960, is the active flavonoid derived from the seeds of milk thistle (*Silybum marianum*) and is most well-known for its use with liver disorders [175]. To our knowledge, silymarin has only been studied in rodent models of β-cell ablation and consequent hyperglycemia (STZ, alloxan, and partial pancreatectomy). The results from these studies showed significant improvements in glucose control and circulating insulin levels in animals treated with silymarin compared to controls [176–179]. These outcomes are promising and suggest that silymarin may elicit beneficial effects in other rodent models of T1DM and T2DM. Silymarin as a co-therapy in a small cohort of individuals with T2DM showed promising results [180], and these findings should be expanded to a larger cohort to determine if silymarin could be used as a potential adjuvant therapy in T2DM.

### In Vivo Studies

In alloxan-exposed Wistar rats (150 mg/kg, single injection), silymarin treatment (200 mg/kg/day, oral gavage) for 9 weeks after onset of diabetes completely attenuated the alloxan-dependent hyperglycemia within 1 week of administration. Consistent with these results, the alloxan-dependent decrease in serum insulin levels was also prevented. Furthermore, assessment of pancreatic tissue showed that silymarin protected against alloxan-induced morphological damage to pancreatic islets and β-cells [176]. Silymarin supplementation (50 mg/kg/day, oral gavage) for 28 days in Wistar rats exposed to STZ (50 mg/kg, single injection) significantly decreased hyperglycemia compared to diabetic controls. Silymarin administration to diabetic rats increased insulin production, and although β-cell number improved in the silymarin group compared to diabetic animals, there still remained a 50% decrease when compared to non-diabetic controls [179].

In partially pancreatectomized Wistar rats (60%) treated with silymarin (200 mg/kg/day, oral gavage) for 9 weeks, serum concentrations of glucose were improved relative to untreated pancreatectomized animals. Serum insulin levels were also increased in silymarin-treated animals, and by day 42 of the study, insulin was more than twice the level of control animals prior to beginning intervention [178]. Similarly, 9 weeks of silymarin treatment (200 mg/kg/day) in partially (60%) pancreatectomized rats improved fasting serum glucose to concentrations seen in control animals, increased serum insulin concentrations beyond the normal control animals, and significantly increased β-cell proliferation compared to control pancreatectomized rats [177].

Although studies are limited, silymarin use has the potential to confer beneficial effects in humans. In male and female Iranian patients with T2DM, silymarin treatment (600 mg/day) for 4 months, in combination with conventional therapy (metformin and glibenclamide), significantly decreased FBG and HbA1c, when compared to the placebo group. However, there was no observable difference in fasting insulin levels [180].

### In Vitro Studies

In MIN6N8a cells, an SV40 T-transformed insulinoma cell line derived from NOD mice, silymarin treatment (50 μg/mL for 48 h) in the presence of inflammatory cytokines (TNFα, IFNγ, and IL-1β) ameliorated cytokine-induced cell death, suppressed NO production, inhibited iNOS expression, inhibited ERK1/2 phosphorylation, and attenuated NF-κB activation [181]. In HIT-T15 cells treated with silymarin (25–500 μM for 2 h), the lower concentrations (25–100 μM) increased GSIS. However, GSIS was diminished with concentrations above 100 μM due to decreased cellular viability. Incubation of HIT-T15 cells with silymarin did not alter basal insulin secretion or insulin content. Additionally, in the silymarin-treated HIT-T15 cells, lower concentrations of silymarin (1–50 μM) were ineffective at suppressing the endogenous peroxide concentrations, while higher concentrations (100–500 μM) dose-dependently suppressed endogenous peroxide concentrations. However, the higher concentrations needed to suppress peroxide concentrations also induced apoptosis [182].

Silymarin increased the expression of insulin and Pdx1 in the pancreas of partially pancreatectomized rats [177]. Additionally, silymarin upregulates the expression and immunolabeling of Nkx6.1 in the pancreas of male Wistar rats following a partial pancreatectomy [178], a gene that plays a critical role in differentiating and maintaining β-cells, as well as maintaining insulin transcription levels. Currently, how silymarin upregulates Nkx6.1 is debated. Silymarin-dependent upregulation of Nkx6.1 may be due to increased expression of Pdx1 [177]. However, Soto et al. suggest that silymarin can increase Nkx6.1 expression directly and independently of Pdx1 [178]. For now, further evidence is needed to fully elucidate this mechanism. Silymarin also inhibits PDE-mediated breakdown of cAMP in HIT-T15 insulinoma cells, suggesting that increased and prolonged intracellular concentration of cAMP may enhance insulin exocytosis [182].

## Ursolic Acid

Ursolic acid, a pentacyclic triterpene first discovered in 1920, is found in several plants including apples, basil, cranberries, thyme, and many more, and is the major component of some traditional medicinal herbs [183] (Table 3). Ursolic acid has been shown to have anti-inflammatory properties, lungs, kidneys, liver, and brain protective effects, skeletal muscle anabolic effects, ability to reduce osteoporosis, and have anti-microbial properties [184]. To our knowledge, ursolic acid has not been studied in genetic rodent models of T1DM and T2DM. However, in models of induced hyperglycemia (STZ) and glucose intolerance (HFD), ursolic acid has been shown to exert positive effects on circulating levels of insulin and glucose [185–188]. Furthermore, studies in patients with metabolic syndrome have also shown anti-diabetic effects [189].

### In Vivo Studies

In ICR mice exposed to STZ (200 mg/kg, single injection), ursolic acid supplementation (0.5 g/kg of diet) in a high-fat diet (37% kcal from fat) for 4 weeks showed a significant, albeit modest, reduction in FBG concentration at 2 weeks. This phenotype was preserved until the end of the 4-week study. Ursolic acid supplementation also showed modest, yet significant, improvements in both glucose and insulin tolerance. These physiological changes were accompanied by an increase in plasma levels of both insulin and C-peptide compared to diabetic mice. Although pancreatic insulin content was enhanced with ursolic acid, it remained three times lower than non-diabetic control levels. Staining for insulin-positive β-cells followed a similar pattern [185].

In Wistar rats exposed to STZ (50 mg/kg, single injection), 28 days of ursolic acid treatment (50 mg/kg/day, oral gavage) reduced blood glucose with similar efficacy as glimepiride. Although ursolic acid treatment significantly elevated plasma insulin levels compared to diabetic control animals, circulating levels were not completely restored to non-diabetic control values as observed with glimepiride [186]. Similarly, in diabetic Wistar rats (STZ 30 mg/kg, 2 injections), 14 days of ursolic acid treatment (200 mg/kg/day, i.p. injection) reduced FBG and enhanced pancreatic β-cell number [187].

Using a glucose overload protocol (4 mg/kg), Wistar rats treated with ursolic acid (1.0 mg/kg, single oral gavage) 30 min before the overload displayed improved glucose clearance compared to control rats. This glucose-lowering effect of ursolic acid was accompanied by increased output of insulin 15 min after glucose overload [190]. A significant drop in the number of cytoplasmic vesicles was detected in β-cells of the ursolic acid-treated group after 5, but not 20, min demonstrating that this compound mediates its effect on the first phase of insulin secretion [190].

In high-fat-fed (60% fat kcal) C57BL/6 mice, ursolic acid supplementation (0.5 g/kg of HFD) for 12 weeks led to complete normalization of HF diet-induced glucose intolerance and prevented a high-fat diet-induced loss in insulin content, as assessed by immunofluorescence staining of pancreatic tissue sections [188].

From a clinical perspective, male and female individuals diagnosed with metabolic syndrome given oral ursolic acid for 12 weeks (150 mg/day, oral capsule) showed a reduction in FBG and improved insulin sensitivity, assessed by the Matsuda Index, when compared to the individuals on the placebo regimen [189].

### In Vitro Studies

From a mechanistic viewpoint, ursolic acid upregulates SIRT1 abundance in pancreatic tissue of diabetic Wistar rats [187]. Increased SIRT1 abundance may mediate the effects of ursolic acid through numerous pathways. In INS-1 and MIN6 cells, SIRT1 has been shown to be a positive regulator of insulin secretion by repressing UCP-2 and elevating ATP concentrations [191]. Adenoviral overexpression of SIRT1 in RIN cells has been shown to dampen β-cell inflammation via suppression of NF-κB signaling [173].

## Wedelolactone

Wedelolactone, an organic chemical compound classified as a coumestan, was first isolated in 1956 from *Eclipta alba*, also known as a false daisy [192] (Table 3). Wedelolactone has been shown to reduce FBG and HbA1c levels in two chemically induced models of diabetes, STZ and alloxan [193, 194]. Furthermore, wedelolactone has been shown to have anti-inflammatory actions in the islet in vivo and in vitro [195].

### In Vivo Studies

Wedelolactone treatment (25 mg/kg/day, oral gavage) for 15 days in diabetic Wistar rats (STZ given at 70 mg/kg, single injection) reduced FBG and HbA1c, and improved circulating insulin and C-peptide levels. This intervention also attenuated the decline in plasma anti-oxidants GPx and CAT seen in diabetic control animals [193]. Wedelolactone also dampens the generation of AGEs [193], which are known to reduce insulin secretion and cause mitochondrial dysfunction and β-cell damage [196].

Alloxan-induced diabetic Wistar rats (150 mg/kg, single injection) treated with a suspension of powdered *Eclipta alba* leaves (2 or 4 g/kg/day, oral gavage) for 60 days was shown to be as effective as glibenclamide in lowering blood glucose and HbA1c levels compared to the diabetic control group [194]. Additionally, in an IL-1β-driven transgenic model of chronic islet inflammation in zebrafish, wedelolactone treatment (30 μM, in their embryonic water) significantly improved FBG, reduced immune cell infiltration, and reduced NF-κB activation [195].

### In Vitro Studies

In isolated islets from CD1 mice, high-dose wedelolactone treatment (1, 5, and 10 μM for 48 h) reduced inflammatory cytokine (IFNγ, TNFα, IL-1β, 20 h) induced caspase-3/7 activation. Wedelolactone also partially attenuated cytokine-induced NO production and iNOS expression. In human islets, wedelolactone treatment also reduced cytokine-induced caspase-3/7 activity [195].

## Conclusion

Herein we have provided a comprehensive review on the use of various botanicals, extracts, and purified compounds and their impact on islet beta-cell mass and function, a topic highly relevant to metabolic disease. With increasing rates of obesity and diabetes worldwide, it is important to investigate all possible therapeutic options and opportunities. The wide variety of plant-derived natural products is one such possibility that, after rigorous research is conducted, may lead to novel first-line and/or adjuvant pharmacologic options to combat these major public health problems.

Indeed, plant extracts have been used for centuries for medicinal benefit, including for treatment of diabetes mellitus [197]. Remarkably, many therapies, including insulin injection, were originally administered to diabetic patients without a complete understanding of their physiological mechanism(s) of action. Likewise, the investigation into the endocrine and biochemical actions of herbal-based approaches has led to discoveries of specific small molecules, including the biguanide compounds (now used as the diabetes drug metformin). These extensively prescribed drugs arose from studies showing that these natural products elicit robust ability to control blood glucose, tissue sensitivity to insulin, and thus after synthetic chemical refinement have developed into current use in modern medical practice.

In summary, the field of botanicals offers opportunities for further investigation, such as purification of compounds from complex botanical extracts with the goal of isolating specific activities that have direct medical benefits (e.g., glucose-lowering or hormone-sensitizing properties). Further, whether herbal blends will provide synergistic benefit over an individual single plant extract still requires more rigorous studies [198]. These and other possibilities provide options for future experimental avenues to address therapeutic potential while also investigating the quantity of extract (e.g., micromolar to nanomolar efficacy) needed for a salutary outcome.

## Figures and Tables

**Table 1:** Chemical name and molecular structure for the botanical compounds berberine, capsaicin, cinnamaldehyde, conophylline, curcumin, ECGC, and genistein

Botanical Compounds		
Botanical	Chemical Name	Molecular Structure
Berberine	5,6-Dihydro-9,10-dimethoxybenzo[g]-1,3-benzodioxolo[5,6-a]quinolizinium	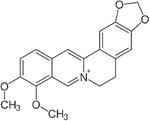
Capsaicin	(6*E*)-*N*-[(4-Hydroxy-3-methoxyphenyl)methyl]-8-methylnon-6-enamide	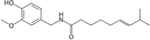
Cinnamaldehyde	2E)-3-Phenylprop-2-enal	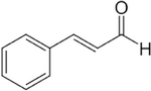
Conophylline	dimethyl (2*R*,6*R*,11*R*,13*S*,14*S*,23*S*,24*S*,25*S*,36*R*,39*R*,40*R*)-14,25-diethyl-24,33-dihydroxy-31,32-dimethoxy-12,22-dioxa-1,9,18,29-tetrazadodecacyclo [23.13.1.1^6,9^.0^2,23^.0^3,21^.0^5,19^.0^6,17^.0^11,13^.0^28,36^.0^30,35^.0^36,39^.0^14,40^] tetraconta-3,5(19),16,20,27,30,32,34-octaene-16,27-dicarboxylate	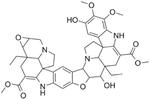
Curcumin	(1*E*,6*E*)-1,7-Bis(4-hydroxy-3-methoxyphenyl)hepta-1,6-diene-3,5-dione	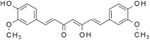
		
EGCG	[(2R,3R)-5,7-dihydroxy-2-(3,4,5-trihydroxyphenyl) chroman-3-yl] 3,4,5-trihydroxybenzoate	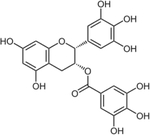
Genistein	5,7-Dihydroxy-3-(4-hydroxyphenyl)chromen-4-one	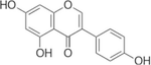

**Table 2:** Chemical name and molecular structure for the botanical compounds gingerol, gymnemic acid, kinsenoside, nymphayol, and paeoniflorin.

Botanical Compounds		
Botanical	Chemical Name	Molecular Structure
Gingerol	S)-5-Hydroxy-1-(4-hydroxy-3-methoxyphenyl)-3-decanone	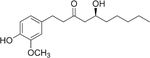
Gymnemic Acid	(2S,3S,4S,5R,6R)-6-[[(3S,4R,4aR,6aR,6bS,8S,8aR,9R,10R,12aS,14aR,14bR)-8,9-dihydroxy-4,8a-bis(hydroxymethyl)-4,6a,6b,11,11,14b-hexamethyl-10-[(E)-2-methylbut-2-enoyl]oxy-1,2,3,4a,5,6,7,8,9,10,12,12a,14,14a-tetradecahydropicen-3-yl]oxy]-3,4,5-trihydroxyoxane-2-carboxylic acid	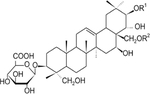
Kinsenoside	(4R)-4-[(2R,3R,4S,5S,6R)-3,4,5-trihydroxy-6-(hydroxymethyl)oxan-2-yl]oxyoxolan-2-one	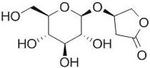
Nymphayol	(3*S*,8*S*,9*S*,10*R*,13*R*,14*S*,17*R*)-17-[(2*R*)-hexan-2-yl]-10,13-dimethy-l-2,3,4,7,8,9,11,12,14,15,16,17-dodecahydro-1*H-*cyclopenta[a]phenanthren-3-ol	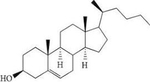
Paeoniflorin	[(1*R*,2*S*,3*R*,5*R*,6*R*,8*S*)-6-hydroxy-8-methyl-3-[(2*S*,3*R*,4*S*,5*S*,6*R*)-3,4,5-trihydroxy-6-(hydroxymethyl)oxan-2-yl]oxy-9,10-dioxatetracyclo[4.3.1.0^2,5^.0^3,8^]decan-2-yl]methyl benzoate	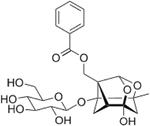

**Table 3:** Chemical name and molecular structure for the botanical compounds resveratrol, quercetin, silymarin, ursolic acid, and wedelolactone

Botanical Compounds		
Botanical	Chemical Name	Molecular Structure
Quercetin	2-(3,4-dihydroxyphenyl)-3,5,7-trihydroxy-4*H*-chromen-4-one	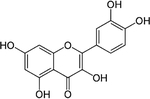
Resveratrol	5-[(E)-2-(4-hydroxyphenyl) ethenyl]benzene-1,3-diol	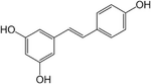
Silymarin	(2R,3R)-3,5,7-trihydroxy-2-[(2S,3S)-3-(4-hydroxy-3-methoxyphenyl)-2-(hydroxymethyl)-2,3-dihydro-1,4-benzodioxin-6-yl]-2,3-dihydrochromen-4-one	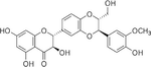
Ursolic Acid	(1S,2R,4aS,6aR,6aS,6bR,8aR,10S,12aR,14bS)-10-hydroxy-1,2,6a,6b,9,9,12a-heptamethyl-2,3,4,5,6,6a,7,8,8a,10,11,12,13,14b-tetradecahydro-1H-picene-4a-carboxylic acid	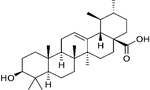
Wedelolactone	1,8,9-Trihydroxy-3-methoxy-6H-[1]benzofuro[3,2-c]chromen-6-one	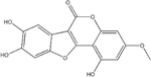

## Data Availability

There were no original data reported in this manuscript.

## Abbreviations

AGEs: Advanced glycation end-products
AMPK: AMP-activated protein kinase
ATP: Adenosine triphosphate
BCL-2: B-cell lymphoma 2
BrdU: Bromodeoxyuridine
Ca^2+^: Calcium
cAMP: Cyclic AMP
CAT: Catalase
CK20: Keratin 20
COX: Cyclooxygenase
COX-2: Cyclooxygenase-2
CRP: C-reactive protein
DCFHA-DA: Dichlorodihydrofluorescein diacetate
DPP4: Dipeptidyl peptidase-4
EGCG: Epigallocatechin gallate
ERK: Extracellular signal-regulated kinases
FBG: Fasting blood glucose
FFA: Free fatty acid
Foxo1: Forkhead box protein O1
Gck: Glucokinase
GDM: Gestational diabetes mellitus
GLP-1: Glucagon-like peptide-1
GLUT2: Glucose transporter 2
GLUT4: Glucose transporter 4
GPx: Glutathione peroxidase
GSH: Glutathione
GSH-Px: Glutathione peroxidase
GSIS: Glucose-stimulated insulin secretion
GSP: Glycosylated serum protein
GST: Glutathione S-transferase
GTT: Glucose tolerance test
HbA1c: Glycated hemoglobin
HFD: High-fat diet
HFHS: High-fat high-sucrose
HNF-1a: Hepatic nuclear factor homeobox A
HOMA-IR: Homeostatic model assessment for insulin resistance
HOMA-β: Homeostatic model assessment of β-cell function
i.g.: Intragastric
i.p.: Intraperitoneal
_i_Ca^2+^: Intracellular calcium
IFNγ: Interferon-γ
IL-10: Interleukin-10
IL-1β: Interleukin-1β
IL-6: Interleukin-6
iNOS: Inducible nitric oxide
ipGTT: Intraperitoneal glucose tolerance test
IRS2: Insulin receptor substrate 2
IV: Intravenous
IκB: Inhibitor of nuclear factor kappa B
IκBα: Nuclear factor of kappa light polypeptide gene enhancer in B cells inhibitor-α
JAK: Janus kinase
JNK: C-Jun N-terminal kinases
K_ATP_: ATP-sensitive potassium channel
K_V_: Voltage-gated potassium channel
MAPK: Mitogen-activated protein kinase
MCP-1: Monocyte chemoattractant protein-1
MDA: Malondialdehyde
MPO: Myeloperoxidase
mTOR1: Mechanistic target of rapamycin complex 1
NF-κB: Nuclear factor kappa-light-chain-enhancer of activated B cells
Nkx2.2: Homeobox protein Nkx-2.2
Nkx6.1: Homeobox protein Nkx-6.1
NO: Nitric oxide
OGTT: Oral glucose tolerance test
PARP: Poly (ADP-ribose) polymerase
PBG: Post-prandial blood glucose
PDE: Phosphodiesterase
Pdx1: Pancreatic and duodenal homeobox 1
PGE2: Prostaglandin E2
PI3K: Phosphoinositide 3-kinase
PKA: Protein kinase A
PPARγ: Peroxisome proliferator-activated receptor gamma
ROS: Reactive oxygen species
SIRT1: Sirtuin 1
SOD: Superoxide dismutase
SQ: Subcutaneous
STZ: Streptozotocin
T1DM: Type 1 diabetes mellitus
T2DM: Type 2 diabetes mellitus
TEM: Transmission electron microscopy
TFAM: Mitochondrial transcription factor A
TG: Triglyceride
TNFα: Tumor necrosis factor-α
TRPA1: Transient receptor potential ankyrin 1
TRPV1: Transient receptor potential vanilloid 1
UCP2: Uncoupling protein 2
ZDF: Zucker diabetic fatty rat
